# What is nursing professionalism? a concept analysis

**DOI:** 10.1186/s12912-022-01161-0

**Published:** 2023-02-07

**Authors:** Huili Cao, Yejun Song, Yanming Wu, Yifei Du, Xingyue He, Yangjie Chen, Qiaohong Wang, Hui Yang

**Affiliations:** 1 Nursing College of Shanxi Medical University, Taiyuan, 030001 Shanxi People’s Republic of China; 2 Linfen Hospital Affiliated to Shanxi Medical University (Linfen People’s Hospital), Linfen, 041000 Shanxi People’s Republic of China; 3 The Third Peoples Hospital of Taiyuan, Taiyuan, 030001 Shanxi People’s Republic of China; 4 The First Hospital of Shanxi Medical University, Taiyuan, 030001 Shanxi People’s Republic of China

**Keywords:** Nursing professionalism, Concept analysis, Attributes, Antecedents, Consequences

## Abstract

**Background:**

Nursing professionalism plays an important role in clinical nursing. However, a clear conceptual understanding of nursing professionalism is lacking.

**Method:**

Walker and Avant’s strategy was used to analyse the concept of nursing professionalism. We searched electronic databases, including PubMed, Scopus, and CINAHL, for studies published from 1965 to 2021. Quantitative or qualitative studies published in English that focused on nursing professionalism were included in the study.

**Results:**

The three attributes of nursing professionalism are multidimensional, dynamic, and culture oriented. Based on the analysis, nursing professionalism is defined as providing individuals care based on the principles of professionalism, caring, and altruism.

**Conclusions:**

This study offers a theoretical definition and conceptual model of nursing professionalism that may be applied to develop standardized assessment tools or nursing professionalism training programs.

## Introduction

The COVID-19 outbreak has exposed deficiencies in the underinvestment of the global health system, including the shortage in nursing resources and nursing staff, and a similar situation is noted in China (https://www.icn.ch/news/investing-nursing-and-respecting-nurses-rights-key-themes-international-nurses-day-2022). An unbalanced number of nurses and patients, high work pressure, lack of social occupational identity and other reasons have led to job burnout, low job satisfaction, and even the resignation of many nurses. Research has also shown that the lack of nursing professionalism adversely affects patient care and patient outcomes [1]. Ohman [2] pointed out that lower levels of professionalism may cause negative outcomes, such as turnover and attrition and lower productivity.

In recent years, researchers have tried to solve the above problems through professionalism.

However, nursing professionalism plays a more important role in clinical nursing. Some studies have shown that professionalism can improve the professional knowledge and skills of nurses and ameliorate reductions in institutional productivity and quality [3]. Higher levels of professionalism can improve nurses’ autonomy and empowerment, increase their recognition and facilitate organizational citizenship behaviours, establish nursing care standards and even improve quality services [4, 5].

Nursing professionalism has been discussed for several decades. Hall (1968) developed the Professionalism Inventory Scale [6]. Miller et al [7] (1993) first specified the 9 standards criteria of nursing professionalism (educational background; adherence to the code of ethics; participation in the professional organization; continuing education and competency; communication and publication; autonomy and self-regulation; community service; theory use, development, and evaluation; and research involvement.). Yeun et al. (2005) summarized five themes regarding nurses’ perceptions of nursing professionalism: self-concept of the profession, social awareness, professionalism of nursing, the roles of nursing services, and originality of nursing [8]. Yoder defined nursing professionalism based on six components: acting in the patients’ interests; showing humanism; practising social responsibility; demonstrating sensitivity to people’s cultures and beliefs; having high standards of competence and knowledge; and demonstrating high ethical standards [9]. Although some researchers have explored the concept of professionalism. How can professionalism be evaluated in nursing clinical practice? Few studies have shown a clear conceptualization of nurses’ professionalism [10, 11]. To nurture nursing professionalism, the concept of professionalism must be clarified.

Given that the meaning of professionalism varies across time, contexts, or cultures, it is difficult to define, quantify or measure professionalism [12, 13]. The operational definition of nursing professionalism in studies has shortcomings. Sullivan et al. [14] found professionalism to be a multidimensional concept, but some papers have addressed only one dimension, such as values [15] or behaviours [16]. Moreover, professionalism is considered a complex concept. The links and dynamic processes between these different inner characteristics have not been included in the concept. Thus, a comprehensive definition of nursing professionalism, including its characteristics and the relations between them, is necessary.

Recognizing and understanding the concept of nursing professionalism may be an essential step towards providing quality care for people. It may also provide more information for further developing nursing professionalism for nurses.

## Methods

### Method of concept analysis

Walker and Avant’s method used linguistic philosophy techniques to contribute to the philosophical understanding of a concept [17]. The W & A method is considered a mark of the positivist paradigm, which views the concept as a stable factor that can be reduced or extracted from its context of application [18]. This study used Walker and Avant’s method, which assumes that nursing professionalism is a relatively mature and stable concept (numerous studies on nursing professionalism have been published to date). This approach to conceptual analysis, although not perfect, is helpful in clarifying the concept of nursing professionalism.

Using the structured method of Walker and Avant enables conceptual clarity to be obtained based on an inductive identification of the concept’s attributes, antecedents and consequences. The concept analysis helps to clarify meanings and develop operational definitions, considering evidence from a wide range of information resources for further research or clinical practice [17, 19]. These features make this method particularly useful for the analysis of the concept of ‘nursing professionalism’. The conceptual attributes as well as antecedents and consequences are based on the research team's analysis of the literature using Walker and Avant’s strategy and are not the product of a priori theoretical categories.

Walker and Avant’s [17] eight-step method includes the following: 1) selecting a concept; 2) determining the aims or purposes of analysis; 3) identifying all uses of the concept; 4) determining the defining attributes of the concept; 5) constructing a model case; 6) constructing borderline, contrary, invented, and illegitimate cases; 7) identifying antecedents and consequences; and 8) defining empirical references.

### Selection criteria

The inclusion criteria were as follows: related to the concept of nursing professionalism; included nurse professionalism, nursing spirit, or nurse spirit; written in the English language; qualitative, quantitative, mixed methods or systematic reviews; published between 1965 and 2021 (when professionalism was first introduced by nursing in 1965); and published in books or dictionaries. We excluded articles published in nonpeer reviewed journals, editorials and letters to the editor.

### Data sources

We searched several online databases, including PubMed, Scopus, and CINAHL, for articles published from 1965 to 2021. We searched the words that appear in the title, abstract, and keyword section of the studies.

#### PUBMED

(((((((((Nursing professionalism[Title]) OR (Nursing professionalism[Title/Abstract])) OR (Nurse professionalism[Title])) OR (Nurse professionalism[Title/Abstract])) OR (Nursing spirit[Title])) OR (Nursing spirit[Title/Abstract])) OR (Nurse spirit[Title])) OR (Nurse spirit[Title/Abstract])).

#### CINAHL

TI Nursing professionalism OR AB Nursing professionalism OR TI Nurse professionalism OR AB Nurse professionalism OR TI Nursing spirit OR AB Nursing spirit OR TI Nurse spirit OR AB Nurse spirit.

#### Scopus

TITLE-ABS-KEY (Nursing professionalism) OR TITLE-ABS-KEY (Nurse professionalism) OR TITLE-ABS-KEY (Nursing spirit) OR TITLE-ABS-KEY (Nurse spirit).

Any quantitative or qualitative studies published in English focusing on nursing professionalism were included in the study. Two researchers independently screened titles and abstracts to determine the selection criteria for electronic retrieval and application. The study was included only when both researchers agreed that the study met the inclusion and exclusion criteria. If the two researchers’ judgements were different, a third person was consulted to resolve the issue. Researchers identified the different usages of the concept and systematically recorded the characteristics of the concept that appeared repeatedly [17].

**Fig.1:**
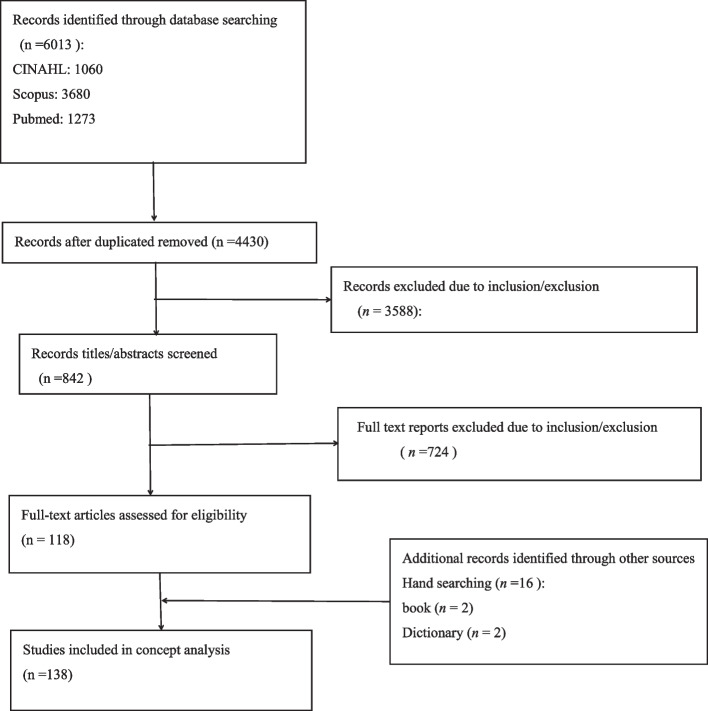
Flowchart of the study selection process of the concept analysis

**Fig. 2:**
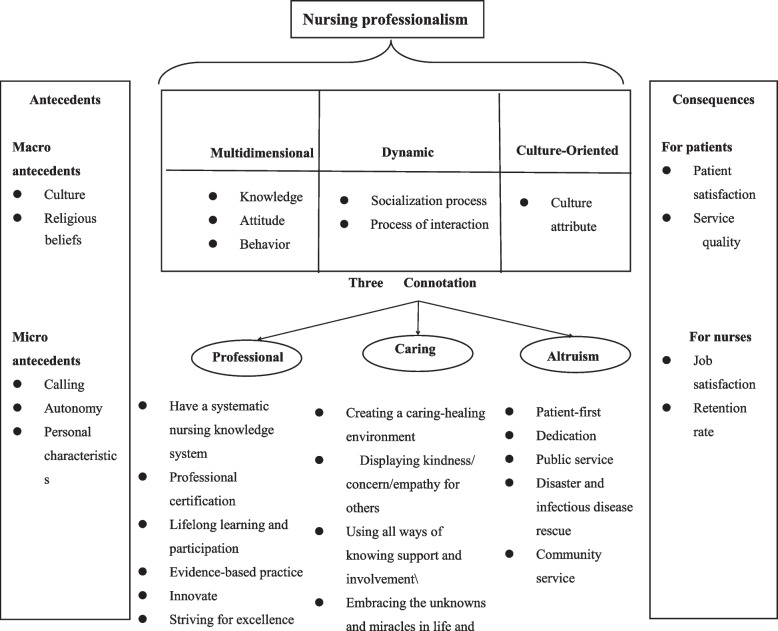
Antecedents, attributes, and consequences of nursing professionalism

We used definitions and examples in the systematic record (Table 2) to define a cluster of antecedents, attributes and consequences (Figs. 1 and 2) frequently associated with the concept [20].

## Results

We identified 6013 studies on nursing professionalism. After excluding duplicates, irrelevant studies, studies that were not original scientific studies or articles, and studies published in languages other than English, 138 studies were selected for analysis. Tables 1 and 2 show some typical literatures used in this study.

**Table 1:** The attributes of the nursing professionalism

Attributes	Nursing professionalism	Author	Country
**Multidimensional**	Knowledge	Fogarty, T. J., et al. (2001) [21]	USA
	Attitude	Wynd C. A. (2003) [22] Hall, R. H.. (1968) [6] Takada, N., et al. (2021) [23]	USA USA Japan
	Behaviours	Schwirian P.M. (1998)? [11] Miller, B. K.. (1988) [24]	USA USA
**Dynamic**	Socialization process	Hinshaw, A.S. (1976) [25]	USA
	process of interaction	Swick H. M. (2006) [12] Dehghani, A(2016) [26]	USA Iranian
**Culture-Oriented**	Cultural attribute of nursing professionalism	Chandratilake, M., et al.(2012) [27] Jin P. (2015) [28]	UK China

**Table 2:** The connotations of nursing professionalism

Connotation	Nursing professionalism	Author	Country
**Professional**	Have a systematic nursing knowledge system	Freidson,(2001) [29] Hinshaw, A. S.(1987) [1]	USA USA
	Professional certification	Lamonte M. (2007) [30] Stucky, C. H., & Wymer, J. A. (2020) [31]	USA USA
	Lifelong learning and participation	Hinshaw, A. S.et al,. (1987) [1] Karadağ, A.,et al.(2007) [32]	USA Turkey
	Evidence-based practice	Cornett B. S. (2006) [33]	USA
	Innovate	Shen et al. (2021) [34]	China
	Striving for excellence	Boehm, L. M.,et al. (2020) [35]	USA
**Caring**	Creating a caring-healing environment	Watson, J. (1988) [36]	USA
	Displaying kindness/ concern/empathy for others	Collins, H., (2014) [37] Papastavrou E., et al. (2011); [38] Jooste, K., (2010), [39]	UK Greek South Africa
	Using all ways of knowing support and involvement	Watson, J. (1988) [36]	USA
	Embracing the unknowns and miracles in life and practicing loving	Watson, J. (1988) [36]	USA
**Altruism**	Patient-first	Kubsch, S, et al. (2021) [40]	USA
	Dedication	Fernandez, R., et al. (2020) [41] Zhang, M, et al.(2021) [42] Goldie J. (2013) [43]	Australia China UK
	Public service	Riley, J. M etal (2010) [44]	UK
	Disaster and infectious disease rescue	McDonald L. (2014) [45] Liu, Q., et al. (2020) [46]	Canada China
	Community service	Kim-Godwin, Y. S(2010) [47]	USA

### Uses of the concept

#### Dictionary definitions of the concept

The Merriam-Webster Dictionary defines professionalism as ‘the conduct, aims, or qualities that characterize or mark a profession or a professional person’ [48], whereas the Cambridge Dictionary [49] defines professionalism as ‘the combination of all the qualities that are connected with trained and skilled people’. These definitions are generic and difficult to use to clarify the factors involved in nursing professionalism.

#### Definitions of the concept reported in the literature

Hwang et al. [50] defined professionalism as commitment to a profession and professional identity level. Health-care workers demonstrate professionalism through attitudes, knowledge, and behaviours, which reflect approaches to the regulations, principles, and standards underlying successful clinical practices [33]. Nursing professionalism reflects the value orientation, concepts of nursing, work attitude and standards of clinical nurses [51].

#### Subconcepts

The Nightingale Spirit, named in honour of the founder of professional nursing, refers to the spirit of altruism, caring, and honesty [52]. In the past, the Nightingale Spirit advocated that nurses are willing to dedicate themselves, but the term currently encompasses more innovation [53]. E-professionalism is defined as evidence provided by digital means, attitudes and behaviours reflects the traditional models of professionalism [54]. Nurses use the internet to communicate about work or daily life, blurring the boundaries between individuals and professions; thus, e-professionalism applies to nurses [55].

### The defining attributes of nursing professionalism

The defining attributes of the concept aim to understand its meaning and differentiate it from other related concepts [17]. The key defining attributes are as follows.

#### Nursing professionalism is multidimensional

Nursing professionalism is a three-dimensional concept based on the knowledge, attitudes, and behaviours that underlie successful clinical practice [33].

##### Knowledge

Professionalism can be conceptualized as a ‘systematic body of knowledge’ with complex configurations of work expertise [21].

##### Attitudes

Professionalism refers to the attitude that represents levels of recognition and commitment to a particular profession [22]. Hall [6] noted that nurses’ attitudes have a high correspondence with the behaviours of the respondent. Measuring professionalism at the cognitive level can be thought of as measuring potential professionalism at the behavioural level. Researchers noted that given the reduced restrictions of environmental constraints, measuring professionalism at the cognitive level may be more precise than measuring it at the behavioural level [23].

##### Behaviours

Nursing professionalism is often described as a set of professional behaviours [11]. Some researchers judge whether nurses exhibit professionalism through their behaviours. Miller [24] (1988) developed the Wheel of Professionalism in Nursing Model. The model is considered a framework for understanding professional behaviours among nurses. Kramer [56] (1975) quantified professionalism by assessing the number of professional books purchased, subscriptions to journals, and the number of articles published.

In addition, the perspective of professional identity formation complements the behaviour-based and attitude-based perspectives on professionalism [57].

#### The formation and development of professionalism are dynamic processes

Nursing professionalism is an inevitable, complex, varied, and dynamic process [58].The professionalism concept is considered ever-changing, replacing static or definitive views [59].

##### Socialization process

Nursing professionalism is instilled through a process of socialization in formal nursing education [25]. Nurses’ socialization process begins with formal, entry-level education to acquire knowledge and skills.

Yeun et al. [8] (2005) discussed the developmental process of nursing professionalism in which the individual’s thoughts and beliefs are formed by socialization factors through perception. These thoughts and beliefs may in turn influence the individual’s professional image or self-concept, thereby influencing nurses’ actions and performance.

##### Process of interaction

The dynamic of professionalism is also reflected in the process of interaction. Dehghani et al. [26]noted that nursing professionalism means the appropriate interaction of the individual and the workplace and the maintenance of interpersonal communication.

#### Culture oriented

One study showed that altruism is an essential element of medical professionalism in Asia or North America but not Europe [27]. In China, medical professionalism was influenced by its longstanding Confucian traditions [28]. Therefore, any definitions of professionalism should match its rooted culture and be validated with respect to the culture and context in which it is applied [60].

### The connotation of nursing professionalism

#### Professional

##### Having a systematic nursing knowledge system

The nursing process is considered a method for solving problems or dilemmas in a logical and scientific manner [11]. Freidson [29] (2001) noted that professionals perform their specialized work only with the required training and experience. Professionals have specific, tacit, almost esoteric knowledge to do their work [61]. Miller et al. [7] considered that a formal university education with a scientific background is critical for professionalism in nursing.

##### Professional certification

Nurses actively seek specialty certification given their personal commitment to the nursing profession [30]. Specialty certification promotes nursing professionalism. When attaining the highest levels of clinical knowledge, nursing professionalism also indicates personal responsibility and dedication to best practices [31].

##### Lifelong learning and participation in continuing education

Due to professional and ethical obligations, nurses should sustain continuous professional growth and development to maintain individual competence. Professional growth in nursing requires lifelong learning. Lifelong learning includes continuing education and self‐study, seeking advanced degrees, etc. [62].

Continuing education is one of the indicators of professionalism. Professionals keep up with the latest developments in the field and partake in continuing education. Additionally, continuing education is as important as other criteria for increasing professionalism in nursing [7, 32]. Ongoing education brings fresh knowledge to health care, consequently leading to more efficient and quality service for people.

##### Evidence-based practice

Evidence-based practice (EBP) is a hallmark of professionalism [33]. Dollaghan [63] (2004) reported that we identify and use the highest quality scientific evidence as an integral part of our efforts to provide the best patient care; EBP is a knowledge base that responds to specific clinical issues in a clear, intelligent, and serious manner while considering clinical practice in the context of the highest-quality scientific evidence available.

##### Innovation

Innovation in nursing helps to improve patient care quality and improve nurses’ job performance [64]. Shen et al. [34] noted that innovative education plays an important role in the professional quality of undergraduate nursing students.

#####  Striving for excellence

Striving for excellence is a requirement and attribute of nursing professionalism. There is a growing need in nursing practice to possess knowledge and skills in quality improvement science, translational research, and implementation science [35]. Clinical nurses have the same responsibilities as nursing scientists.

#### Caring is considered the core attribute of nursing professionalism

The practice of caring is central to nursing [65]. Caring is defined as the moral ideal of nursing [36]. Therefore, caring is an important core attribute of nursing professionalism.

##### Creating a caring-healing environment

Nurses devoted to creating a caring-healing environment embody professionalism. Caring means nurses should create a healing environment at all levels by providing a supportive, protective environment as well as a corrective mental, physical, societal, and spiritual environment for patients. People’s basic needs include a clean environment, comfort measures, safety concerns, and feeling safe or protected [65].

##### Displaying kindness/concern/empathy for others

A nurse is defined as someone caring for the ill within the hospital setting [66]. Caring means showing or having compassion, concern and empathy for others [37]. Caring behaviours are an interactive and mental process between patients and nurses [38]. Displaying kindness and concern for others is shown by love, compassion, support and involvement [39].

##### Using all methods of knowing support and involvement

‘Human problems reside in ambiguity, paradox, and impermanence’. Therefore, suffering, healing, miraculous cures, and synchronicity are all part of knowing support and involvement.

Researchers suggest that nursing comprises Caritas Nursing, Energy Nursing, Transpersonal Nursing, Holistic Nursing, or Contemplative Nursing…… It goes beyond ordinary nursing. Nursing should have higher standards with excellence for caring, healing, and peace in the world. Therefore, caring means using all methods of knowing support and involvement [65].

##### Embracing the unknowns and miracles in life and practising loving

Nursing is a special profession. Nurses confront special circumstances daily and witness people’s struggles with life and death. Everyone has his or her own specific story about his or her experiences and predicaments. Each person seeks his or her own meanings to find inner peace and balance in the midst of fear, doubts, despair, and unknowns. Therefore, the care of nurses is not to blindly sacrifice their own needs but to be a real nurse, embracing the unknowns and miracles in life and caring for patients [65].

#### Altruism

The central tenet of professionalism is to put the needs and best interest of others over self-interests. Altruism is an engagement in caring acts towards others without expecting something in return [67].

##### Patients first

To be altruistic means to put others’ needs before your own. Altruism is the selfless concern for others and doing things with the other person’s well-being in mind [40].

##### Dedication

During pandemics, nurses were considered to have a high sense of duty and dedication to patient care [41]. Front-line nurses perceive high work engagement, especially in self-dedication [42]. Grøthe et al. [43] showed that cancer patients in a palliative unit appreciate nurses who have the most dedication and expertise characteristics.

##### Public service

Due to a strong sense of civic and social responsibility, nurses participate in public service. Nurses volunteer as participants in summer camps, schools, or health-care teams. Nurses are also committed to responding to large-scale crises, such as the terrorist attacks on the World Trade Center in New York, as well as national and international relief efforts, such as tsunamis and Hurricane Katrina [44].

##### Disaster and infectious disease rescue

Individuals involved in providing disaster relief face many challenges, experience fatigue and personal suffering, and encounter numerous personal stories of life and death [45]. Nurses have played a significant role in the fight against infectious diseases such as severe acute respiratory syndrome (SARS) and the coronavirus disease 2019 (COVID-19) pandemic [68]. Nurses are closest with patients. Nurses provide intensive care, regularly assessing and monitoring airways, tubes, medications, and physical therapy. Nurses are also devoted to reducing complications. Nurses assist with daily living activities when patients are unable to care for themselves [46].

##### Community service

In addition, emphasizing professionalism means respecting values and commitment to community service delivery [69].

### Cases

According to Walker and Avant [17], cases help further clarify concepts.

#### Model cases (a real case example)

Model cases help demonstrate all the defining attributes of a concept and helps to better articulate its meaning [17].

MS A is a 63-year-old nursing director. She worked in clinical nursing and management for 42 years. As she progressed from a new nurse to a nursing expert, she gradually poured her enthusiasm (Multidimensional: Attitudes) into nursing work (Dynamic). She believes that the core of nursing professionalism in China is dedication and responsibility (Culture oriented). In 2020, COVID-19 broke out in Wuhan, China. She led a team to Wuhan to provide support (Multidimensional: Behaviours), reflecting the spirit of altruism (Altruism). She actively promoted exchanges and cooperation among disciplines and the development of academic conferences. She guided students to pay attention to practical innovation and develop evidence-based innovations (Professional). Although she is retired, she still imparts knowledge and experience to students everywhere (Multidimensional: Behaviours). She stated that the development of nursing professionalism is very difficult and requires nursing education and role models. (Multidimensional: Knowledge). The role of a nurse is like that of a mother, bringing care to the people (Caring).

#### Borderline cases (a real case example)

Borderline cases provide the examples that contain the most defining attributes of the concept [17].

B is a novice nurse. When working in the infection ward, she was so worried about being infected. She was reluctant to care for patients and wanted to escape from the ward environment. Fortunately, her nurse manager fully understood her situation and helped her adapt to work and reduce her anxiety. B observed that her nurse manager had been helping patients solve problems and giving them comfort and hope. This prompted her to think about what nursing truly means. In 2020, she volunteered to help COVID-19 patients (Altruism).

#### Related cases (a real case example)

Related cases are related to the concept but do not contain all its defining attributes [17].

C is a novice nurse. After graduating from nursing school, he became a nurse in the emergency department. He saw many patients who died or recovered, which made him realize the importance of caring (Caring). He said that emergency nurses need strong professionalism (Multidimensional: Attitudes). He participated in social service activities (Multidimensional: Behaviours), for example, promoting knowledge of cardiopulmonary resuscitation (Altruism) in the community. After working for five years, he returned to school for a master’s degree to help the head nurse conduct nursing research or evidence-based practice (Professional). In his Asian cultural milieu, his is embarrassed about his identity as a male nurse (Culture oriented), but he believes he can do well.

#### Contrary cases (a fictional case example)

A contrary case does not include any defined attributes of the concept [17].

D is a nurse in paediatrics. She disliked nursing when she was a nursing student and even did enjoy communicating with patients (poor dynamics). She was exhausted after work and felt her life was out of balance. One of the values of the hospital where she worked was dedication, which confused her (Poor culture orientation). She considers it unrealistic to require professionalism (Poor nursing professionalism knowledge) and thinks that taking care of new-borns is particularly troublesome (Poor nursing professionalism attitudes), so she is always careless in her work (Multidimensional: poor attitude). D’s child felt ill last week, so she secretly reduced a patient’s medicine (Poor nursing professionalism behaviours) and took the remaining medicine home for her child (lack of altruism). She stopped doing so after her colleagues sensed something strange. One day, a baby kept crying; D reported it to the doctor and did not make further observations (Poor professional). When the shift nurse took over, she observed abnormal limb activity on one side of the child. The child’s family asked the nurse to bear legal responsibility. D said it was no big deal; she no longer wanted to be a nurse (Poor dynamic, professionalism not established).

### Antecedents

Antecedents are events that occur before the intended concept [17].

#### Macro antecedents

##### Culture

Jin [28] suggested that the conceptualization of professionalism is influenced by culture. Employees defined organizational culture underlies an organization’s values and beliefs [70]. Nursing professionalism may be supported by a variety of cultures, so a firm understanding of and personal congruence with each particular culture is essential [71].

##### Religious beliefs

Religiosity is another contributing factor in the cultivation of altruism [72]. Taylor noted that nurses’ job motivation and views of the patient and nursing services are affected by their religious beliefs [73].

#### Micro antecedents

##### Calling

Snizek [74] (1972) reported that devotion to work is a professional value originating from a sense of calling to the field. Liaw et al. [75] (2016) found that nursing students who had caring and compassionate qualities as the most common personal characteristics strongly believed that they were called to nursing.

##### Autonomy

Individuals who pursue excellence in the workplace may be described as motivated and devoted to their work. Attree [76] (2005) noted that nurses’ perceived lack of autonomy over their practice could impact quality of care.

##### Personal characteristics

Nursing professionalism is influenced by various factors, such as educational background, personal interests, professional satisfaction, and professional values [77, 78, 79]. In each country, nurses with higher educational levels may have a higher level of professionalism [22]. Professionalism is thus a trait related to personal character and upbringing [80]. Researchers [81] have demonstrated that professionalism is positively associated with female gender, striving for professional goals, and acceptability. One study found that people’s values tend to shift to emphasize altruism over personal gain as they age [79]. Nursing professionalism is closely associated with personality traits (extraversion, conscientiousness, and agreeableness) [82].

### Consequences of nursing professionalism

Consequences are events or incidents that are the result of the occurrence of a concept [17].

#### Consequences for patients 

Professionalism is one of the decisive factors that critically influences patient satisfaction [50]. Professionalism can also improve practising nurse career development and the quality of service [81].

#### Consequences for nurses

Studies have shown that professionalism and a sense of belonging with colleagues and managers affect the satisfaction [83] and retention rate of nursing students in academic institutions [84]. Izumi et al. [85] (2006) found that good nurses felt pride and happiness in caring for patients closely related to their professionalism.

### Empirical references

As the last step to concept analysis, empirical references can further clarify the concept and facilitate its measurement [17].

#### Hall’s professionalism inventory scale

Hall’s Professionalism Inventory Scale [6] identified five attitudinal attributes of professionalism: (a) use of professional organizations as major referents, (b) belief in public service, (c) self-regulation, (d) a sense of calling to the field, and (e) autonomy. Nursing researchers used Hall’s Professionalism Inventory Scale to measure professionalism in nursing [22, 47]. Snizek [74] (1972) modified the professionalism scale to more closely match the clinical context of nursing and better reflect the professionalism of nursing staff.

#### Kramer’s index of professionalism

Kramer (1974) [86] constructed an index of professionalism that includes indicators of behaviours, such as the number of professional books published, subscriptions to professional journals, hours spent on professional reading, continuing education, participation in professional organizations, number of professional publications, speeches given, committee activity, and participation in research.

#### The behavioural inventory for professionalism in nursing (BIPN)

The Behavioural Inventory for Professionalism in Nursing [7] (BIPN) identifies professional behaviours and values among nurses. The nine categories in the BIPN are (1) educational background; (2) adherence to the code of ethics; (3) participation in the professional organization; (4) continuing education and competency; (5) communication and publication; (6) autonomy and self-regulation; (7) community service; (8) theory use, development, and evaluation; and (9) research involvement.

### Definition of the concept

Based on the present analysis, we define nursing professionalism as follows: ‘Nursing professionalism is a multidimensional concept manifested by the knowledge, attitudes, and behaviours that underlie successful clinical practice. Nursing professionalism is dynamicized through a process of socialization in formal nursing education. This feature is also reflected in the process of interaction. Therefore, nursing professionalism should match its rooted culture.

The connotations of nursing professionalism include professional, caring, and altruism. These connotations are detailed as follows:

#### Professional

- Possesses a systematic nursing knowledge system; professional certification
- Exhibits lifelong learning and participation
- Participates in evidence-based practice
- Demonstrates innovation
- Strives for excellence

#### Caring

- Creates a caring-healing environment
- Displays kindness/concern/empathy for others
- Uses various methods of knowing support and involvement
- Embraces the unknowns and miracles in life and practices loving

#### Altruism

- Patient-first
- Dedication
- Public service
- Disaster and infectious disease rescue
- Community service

A conceptual model of nursing professionalism is shown in Fig. 2.

## Discussion

### Defining the connotation of nursing professionalism

Nursing professionalism has been defined as professional, caring, and altruistic.

Professional values are characteristic of nursing professionalism. Nursing work requires rich knowledge and scientific evidence-based work to improve the quality of nursing services for patients. Nurses need lifelong learning, qualification certification, and participation in academic and practical activities.

Caring is regarded as the core of professionalism. This study suggests creating a caring-healing environment, displaying kindness/concern/empathy for others, employing all methods of knowing support and involvement, embracing the unknowns and miracles in life and practising loving to care for people to obtain high professionalism. This study notes that nursing professionalism emphasizes care for the individual patient and that the nurse does everything possible to create a caring and healing environment for patients. In different health systems worldwide, nurses have incorporated caring about nursing professionalism into everything they do. This characteristic is consistent with Nightingale's view that “Nurses need to be sensitive. A nurse must use her brain, heart and hands to create healing environments to care for the patient’s body, mind and spirit” [87, 88].

Nursing has an altruistic nature, and people interested in helping patients are attracted to this profession [89]. However, some studies have shown that altruistic care is equated with self-sacrifice, self-denial, and unidirectional and unconditional care [90]. Care for a nurse’s own needs is equally important, but nurses should be able to put aside their own needs when required to focus on the needs of others [91]. Nurses should view self-care and altruism as dialectical. Self-realization and providing care for others are not conflicting concepts [92].

### Defining the attributes of nursing professionalism

In this study, we defined nursing professionalism as multidimensional, dynamic, and culture oriented.

Nursing professionalism is a multidimensional concept that includes knowledge, attitudes, and behaviour. Previous studies have defined professionalism as the degree of commitment by individuals to the values and behavioural characteristics of a specific career identity [6, 7]. However, current research on nursing professionalism is mostly single dimensional. The Behavioural Inventory for Professionalism in Nursing (BIPN) is based on Miller’s model and is used to measure professional behaviours among nurses [7]. Hall’s Professionalism Inventory Scale [6] identified five attitudinal attributes of professionalism. This study highlights that it is also necessary to focus on the knowledge dimension of professionalism. Nursing students and nurses should first understand the nursing professionalism that is necessary to become a nurse, which may be the first step in developing professionalism. Nursing students and nurses need to know the values that are necessary to practice the nursing and not have vague impressions. Some studies have shown that nursing students or nurses learn values and norms in informal trainings [93]. Therefore, this study suggests that the development of assessment tools for the knowledge dimension of professionalism is also necessary. Multidimensional evaluation tools are not available for nursing professionalism. Thus, clarifying the multidimensional nature of nursing professionalism will contribute to the development of multidimensional evaluation tools.

Moreover, understanding the dynamics of professionalism is helpful for cultivating nursing professionalism in stages and steps. Inquiries into medical professionalism should be integrated into the culture of social media interaction [94]. Nursing educators and managers should dynamically cultivate nursing professionalism in their interactions.

Differences in the connotation of nursing professionalism are noted in different cultures. This study suggests that the cultivation and evaluation of nursing professionalism need to consider the cultural attributes of different regions and countries.

### Future research directions

- Exploring the antecedents of nursing professionalism can help schools or hospitals cultivate nursing professionalism and develop courses and specific measures.

The macro antecedents of nursing professionalism include culture and religion, and the micro antecedents include calling, autonomy, and personal characteristics. Some researchers have explored methods to cultivate nursing professionalism; for example, role modelling, feedback, group discussions, case-based discussions, reflection, holding ethical rounds, and reports potentially represent more effective methods [95]. Some researchers have tried to enhance professionalism through social media [96]. One of the findings this study is that nursing professionalism is complex and its cultivation difficult. Studies have shown that didactic lectures are ineffective for teaching professionalism [97]. The development of true nursing professionalism requires national advocacy and the immersion of a good professional environment that incorporates professionalism into daily nursing practice. Role modelling is considered an effective method for developing professionalism in nursing [98]. Therefore, this study suggest that studies should be actively conducted to deeply discuss the causes and processes affecting professionalism and to cultivate and intervene at macro and micro levels as well as the key time periods and populations that form professionalism to truly shape the formation of professionalism. Moreover, an environment for building professionalism [99] is very important. Williams [100] (2015) considered that the development of professionalism should begin as early as the first semester of an undergraduate nursing course. One of the themes of nursing students’ professional identity development is ‘doing-learning-knowing-speaking’. Students should develop professionalism in all these areas of nursing practice.

- The relationship between nursing professionalism and health outcomes or nurses’ human resources needs to be further studied.

Our research suggests that the ultimate goal of nursing professionalism is to serve patients with professional knowledge and special professional quality. The public has become increasingly aware of certain possibilities, limitations, and consequences of professionalism. COVID-19 significantly increased the discussion of professionalism and patient outcomes.

Improving professionalism has a positive impact on job satisfaction, professional quality of life, and the willingness to continue in the profession [101, 102, 103]. Therefore, it is important to improve support for nurses, create a good environment for professionalism, and establish a training system for professionalism, thus paving the way to enhance training in professionalism and create opportunities for nurses.

## Implications for nursing management

In April 2020, the World Health Organization (2020) issued the First State of the World’s Nursing 2020 [104]. The report highlighted that nursing professionals are the largest occupational group in the health sector, numbering 27.9 million worldwide. Nurses spend more time with patients than any other health care professionals [105].

Worldwide, nursing professionalism is considered important and associated with expectations. This study clarifies the concept of nursing professionalism and contributes to a framework for developing a theoretical model as well as instruments to measure the concept. A conceptual model of nursing professionalism may increase nurse managers’ insight into nurses’ behaviours and values, creating a good working environment.

Nurse managers should integrate nursing professionalism into their philosophy, mission, and objectives and provide necessary resources, tools, and projects to develop professionalism among nurses. Nurses should cultivate professionalism to provide good nursing services to patients. Further research should explore the relationship between nursing professionalism and patient health outcomes and formulate effective training programs for professionalism.

## Limitations

This conceptual analysis has some limitations. First, research on nursing professionalism published in English may be conducted in different countries and cultures. However, it is also necessary to obtain a more comprehensive and mature concept of the study of different national languages. Second, the lack of research on the combination of all elements of professionalism may lead to overestimation of the impact of these subelements on professionalism. Third, the concept analysis focused on the research process and the researchers’ perspectives, possibly reflecting a lack of other professional understandings of nursing professionalism in medical groups. In addition, the concept analysis included a risk of selection bias, extraction bias, and analysis bias because the study selection process, data extraction, and analysis were all conducted by two researchers. Despite these risk, the studies were all described accurately and systematically.

## Conclusion

Nursing professionalism is one of the important foundations of clinical nursing. It is multidimensional, dynamic, and culture oriented. Based on the analysis, nursing professionalism has been defined as providing people care based on principles of professionalism, caring, and altruism. The definition, attributes, antecedents, consequences, and reference analysis of the experience of nursing professionalism determined in this study provide a theoretical basis for future research. This information can be used to evaluate nursing professionalism, develop assessment tools, or generate theory-based training courses and interventions.

## Data Availability

Data used to support the findings of this study are available from the corresponding author upon request.
